# Growth hormone enhances ovarian response but not live births in DOR patients undergoing PPOS with freeze-all strategy: a retrospective cohort study contrasted with Kuntai capsule

**DOI:** 10.3389/fendo.2026.1897970

**Published:** 2026-08-03

**Authors:** Xiaoju Wan, Min Yu, Xingwu Wu, Zhihui Huang, Jun Tan

**Affiliations:** Reproductive Medicine Center, Jiangxi Maternal and Child Health Hospital, Nanchang, China

**Keywords:** diminished ovarian reserve, freeze-all, growth hormone, IVF/ICSI, Kuntai capsule, PPOS

## Abstract

**Objective:**

The Progestin-Primed Ovarian Stimulation (PPOS) protocol with a freeze-all strategy is increasingly used for diminished ovarian reserve (DOR) patients. While growth hormone (GH) and Kuntai capsule are proposed adjuvants, their efficacy in this specific context remains unclear. This study aimed to determine whether pretreatment with these adjuvants improves live birth rates.

**Methods:**

This retrospective cohort study (2015-2025) included 2, 969 DOR patients undergoing PPOS cycles. Participants were stratified into control (n=1, 665), Kuntai capsule (n=524), and GH (n=778) groups. Propensity score matching (1:1:1) balanced baseline characteristics, yielding 439 patients per group. The primary outcome was oocyte yield; secondary outcomes included embryological parameters, biochemical pregnancy, clinical pregnancy, and live birth. Generalized estimating equations controlled for confounders.

**Results:**

GH significantly enhanced ovarian response versus control: higher peak E_2_ (607 vs. 518 pg/mL, p<0.001), increased oocyte yield (3.0 vs. 2.0, p<0.001), and more available embryos (2.0 vs. 1.0, p=0.002). Biochemical pregnancy was higher with GH (67.4% vs. 57.3%, p=0.006). However, clinical pregnancy (45.7% vs. 44.5%) and live birth rates (27.8% vs. 28.2%) were comparable (p>0.05). GEE confirmed GH was not a predictor of live birth (aOR 0.85, 95% CI 0.54-1.33). Kuntai capsule showed no significant improvement in any ovarian response, embryo quality, or clinical outcomes, including live birth (27.0% vs. 28.2%, p>0.05). Multivariate analysis identified advanced age (>37 years) as the strongest negative predictor of live birth (aOR 0.36, p<0.001).

**Conclusion:**

In DOR patients managed with PPOS and freeze-all, GH pretreatment improves quantitative stimulation metrics and biochemical pregnancy but does not increase clinical pregnancy or live birth. Kuntai capsule demonstrates no beneficial effects across all endpoints. These findings do not support routine use of these adjuvants in this treatment paradigm.

## Introduction

Diminished ovarian reserve (DOR) signifies a critical reproductive health issue characterized by a quantitative reduction in the ovarian follicle pool (1, 2). The diagnosis typically relies on a combination of biomarkers, including elevated basal follicle-stimulating hormone (FSH), decreased anti-Müllerian hormone (AMH) levels, and a low antral follicle count (AFC) (3). DOR is associated with adverse assisted reproductive technology (ART) outcomes (4–6), imposing significant psychological and economic burdens on affected women seeking fertility treatment, and remains a central challenge in reproductive medicine.

For patients undergoing *in vitro* fertilization (IVF) or intracytoplasmic sperm injection (ICSI) in whom a fresh embryo transfer is not planned, the Progestin-Primed Ovarian Stimulation (PPOS) protocol has gained prominence as an effective controlled ovarian stimulation (COS) strategy (7), as it reliably suppresses premature luteinizing hormone (LH) surges and offers practical advantages, including oral administration, reduced monitoring frequency, and lower overall costs (8, 9). Accumulating meta-analytic evidence has further demonstrated the efficacy of the PPOS protocol specifically in DOR patients (10, 11).

Given the suboptimal outcomes often observed in DOR, adjuvant therapies are frequently explored to enhance success. Kuntai capsule, a traditional Chinese medicine approved for conditions related to ovarian function decline, is theorized to nourish the kidney and enrich yin based on its formulation principles (12–15). While some small-scale studies suggest potential benefits on ovarian response and embryo quality in DOR patients undergoing IVF/ICSI, these investigations are limited by sample size, involvement of fresh embryo transfers, and a lack of focus on the specific PPOS context with a freeze-all strategy (16, 17).

Growth hormone (GH), integral to cellular metabolism and development, has also been investigated for its potential role in female reproduction (18, 19). Meta-analyses indicate that GH co-treatment might improve oocyte yield in DOR patients, but the evidence is marked by significant heterogeneity in protocols and patient populations (20, 21). Beyond its potential effects on folliculogenesis, a compelling hypothesis suggests that GH’s primary benefit may be mediated through endometrial receptivity (22). In the PPOS protocol, however, this potential endometrial benefit is completely circumvented, as all embryos are vitrified and transferred in subsequent frozen-thawed cycles.

Therefore, a critical evidence gap exists regarding the efficacy of both Kuntai capsule and GH as adjuvants specifically within the modern PPOS and freeze-all paradigm for DOR. This large-scale, propensity score-matched cohort study was designed to definitively assess whether pretreatment with these adjuvants improves ovarian response and whether such improvements translate into superior clinical outcomes.

## Materials and methods

### Study design and population

This large, retrospective cohort study was conducted at a single reproductive medicine center, enrolling DOR patients who underwent their IVF/ICSI cycle using a PPOS protocol between January 2015 and February 2025. This retrospective study was conducted in accordance with the fundamental principles of the Declaration of Helsinki and was approved by the Reproductive Medicine Ethics Committee of Jiangxi Maternal and Child Health Hospital (Approval No. SZYY-202504). The need for informed consent from participants was waived by the aforementioned ethics committee due to the retrospective nature of the study.

**Figure 1:**
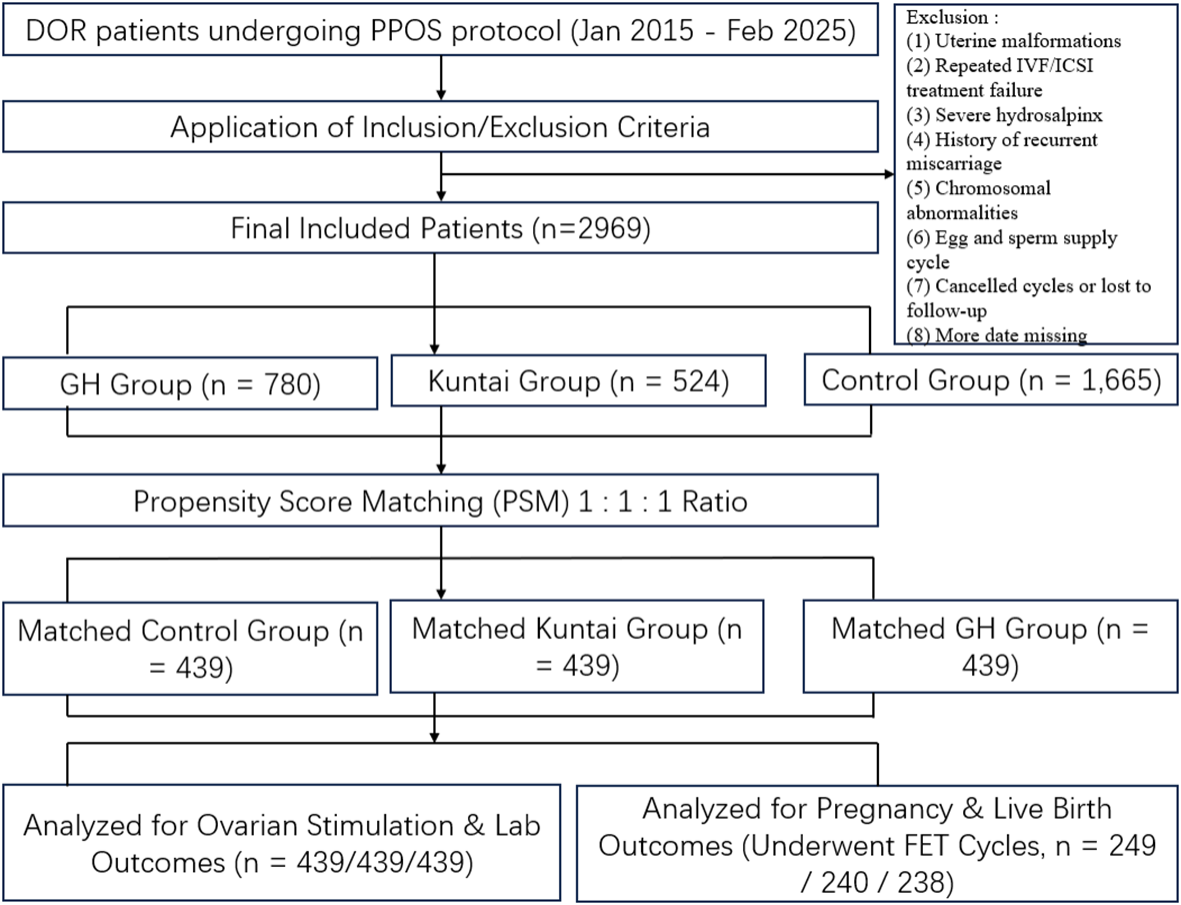
Patient screening, grouping, and analysis flowchart.

DOR was defined by the presence of at least one of the following criteria: AMH < 1.1 ng/ml, AFC < 7, or basal FSH ≥ 10 IU/L. Key exclusion criteria were chromosomal abnormalities, uterine malformations or organic diseases, severe hydrosalpinx, recurrent miscarriage history, donor gamete cycles, loss to follow-up, and cycles cancelled for poor follicular development. Initially, 2, 969 couples were categorized into three groups based on pretreatment: a control group (n=1, 665), a Kuntai group (n=524), and a GH group (n=780), and the research process was illustrated in **Figure 1**.

### COS regimen and pretreatment of Kuntai capsules and GH

All patients in this study underwent the PPOS protocol, which is a commonly used controlled ovarian stimulation strategy for DOR patients in our center. Gonadotropin (Gn) stimulation was initiated on menstrual cycle day 2 or 3 using urinary follicle stimulating hormone (FSH, Lishenbao, Shanghai Lizhu; 150–300 IU/day). Simultaneously, oral medroxyprogesterone acetate (medroxyprogesterone acetate; Zhejiang Xianju; 8–10 mg/day) was administered to prevent LH surges.

Kuntai Group: Patients additionally received oral Kuntai Capsule (Guiyang Xintian, 0.5 g/capsule; 4 capsules, three times daily) from cycle day 3 until oocyte retrieval.

GH Group: Patients received daily subcutaneous recombinant human growth hormone (Changchun Jinsai; 3 IU/day) concurrently with Gn stimulation until the trigger day.

### Egg retrieval, embryo culture and fresh embryo transfer

Triggering was performed with a combination of subcutaneous injection of 0.2 mg of triptorelin acetate (Dabijia, Germany) and intramuscular injection of human chorionic gonadotropin (hCG, Shanghai Lizhu) 2000U when follicular criteria were met (one follicle with a diameter ≥ 18 mm or three follicles with a diameter ≥ 17 mm). Oocytes were retrieved 34–36 hours later via transvaginal ultrasound puncture and fertilized via IVF/ICSI. Embryos were cultured in in G1/G2 (Vitrlife, Sweden) media, with cleavage-stage embryos graded on day 3 and blastocysts on day 5/6. All viable embryos, including both cleavage-stage and blastocyst-stage embryos, were vitrified (RapidVit vitrification method; Vitrolife) for subsequent frozen-thawed embryo transfer (FET).

### Endometrial preparation and frozen-thawed embryo transfer

For frozen-thawed embryo transfer, the endometrium was prepared using one of four protocols, chosen based on the patient’s individual clinical profile:

Natural Cycle: For normal ovulation women. Monitoring began around cycle day 12. Intramuscular progesterone (40–60 mg/day, Zhejiang Xianju) was initiated following the detection of the LH surge.

Artificial Cycle: For women with irregular ovulation. Oral estradiol valerate (Bujiale, Bayer, Germany; 4–8 mg/day) was started on cycle day 2–3. After 12–14 days, if no dominant follicle was observed, endometrial transformation was induced with vaginal progesterone (80 mg/day, Zhejiang Xianju).

Down-regulated Artificial Cycle: For patients with a history of intrauterine adhesion or cesarean section. A long-acting GnRH agonist (leuprorelin acetate, 3.75 mg, Beiyi, Shanghai Lizhu) was administered on cycle day 2–3. After 28 days of down-regulation, the endometrial preparation followed the artificial cycle protocol.

Ovulation Induction Cycle: For patients with natural cycle follicular dysplasia or to shorten preparation time. Letrozole (Furui, Jiangsu Hengrui; 5 mg/day) was administered orally for 5 days starting on cycle day 2–3. From day 10–12, urinary FSH (HMG, Le Baode; Zhuhai Lizhu; 150 IU/day) was added if needed. When the endometrial thickness reached ≥ 8 mm and urinary LH was ≤20 mIU/ml, hCG (4000 IU) was administered. Progesterone supplementation was started after ultrasound-confirmed ovulation.

Either one or two cleavage-stage embryos were transferred on the 4th day of progesterone exposure, or one or two blastocysts were transferred on the 6th day.

### Luteal support and outcome definitions

Luteal support commenced on the day of embryo transfer, consisting of oral dydrogesterone (Duphaston, Abbott, Netherlands; 20 mg/day) combined with vaginal progesterone sustained-release gel (Crinone, Merck Serono, Germany; 90 mg/day). This support was continued until 10 weeks of gestation.

The primary outcome was oocyte yield, chosen to quantitatively assess ovarian response to the adjuvants. Secondary outcomes included embryological and clinical parameters, to evaluate whether improvements in ovarian response translated into genuine reproductive benefits. Primary and secondary outcomes were defined as follows:

Primary Outcome: Number of oocytes retrieved.

Secondary Outcomes:

Oocyte failure rate = (Cycles with 0 retrieved oocytes/Total retrieval cycles) × 100%.

Normal fertilization rate = (Number of 2PN zygotes/Total oocytes retrieved) × 100%.

Normal cleavage rate = (Number of cleavage-stage embryos/Number of normally fertilized oocytes) × 100%.

D3 high-quality embryo rate = (Number of high-quality D3 embryos/Number of cleavage-stage embryos) × 100%.

Available embryo formation rate = (Number of available embryos/Number of cleavage-stage embryos) × 100%.

Unusable embryo rate = (Cycles with no usable embryos/Total retrieval cycles) × 100%.

Biochemical pregnancy was defined as a serum hCG ≥ 5 mIU/ml 14 days after transfer.

Implantation rate = (Number of gestational sacs/Number of embryos transferred) × 100%.

Clinical pregnancy was confirmed by ultrasound visualization of a gestational sac with cardiac activity at 5 weeks post-transfer.

Pregnancy loss rate = (Number of spontaneous or therapeutic abortions/Number of clinical pregnancies) × 100%.

Live birth rate = (Number of live births ≥ 28 weeks/Number of transfer cycles) × 100%.

### Data statistics and analysis

Data were analyzed using R software (version 3.3.4). Continuous variables were tested for normality with the Shapiro-Wilk test. Normally distributed data are presented as mean ± standard deviation and were compared using one-way ANOVA with Tukey’s HSD *post-hoc* test. Non-normally distributed data are expressed as median [interquartile range, IQR] and were compared using the Kruskal-Wallis test with Dunn’s *post-hoc* test (Bonferroni-adjusted p-values). Categorical data are presented as n (%) and were compared using the Chi-square test or Fisher’s exact test, with Bonferroni adjustment for *post-hoc* pairwise comparisons.

To mitigate potential selection bias and balance baseline characteristics (including female age, BMI, basal FSH, AFC, AMH, infertility type, duration, and causes) across the three groups, we employed propensity score matching (PSM) (23). A 1:1:1 matching ratio was used, resulting in 439 well-matched patients per group for the final analysis.

To account for potential data clustering (where some patients contributed more than one treatment cycle), we utilized a Generalized Estimating Equation (GEE) model based on logistic regression to control for confounding factors when analyzing pregnancy outcomes (24). The GEE models were adjusted for treatment group (control, Kuntai, GH), age group (<35, 35–37, >37 years), BMI group (<18.5, 18.5–24, >24 kg/m^2^), endometrial preparation protocol (artificial cycle, downregulated artificial cycle, or other), infertility cause (tubal factor, male factor, or other), number of embryos transferred, and embryo stage (cleavage-stage vs. other).

Given the retrospective nature of this study, no *a priori* sample size calculation was performed. Instead, we conducted a *post-hoc* power analysis for the primary outcome (oocyte yield) and key secondary clinical outcomes (clinical pregnancy, pregnancy loss, and live birth) to assess whether the observed differences could be reliably detected with the current sample size.

A p-value < 0.05 was considered significant.

## Results

### Baseline and cohort characteristics before and after matching

The patient selection, grouping, and propensity score matching workflow is illustrated in **Figure 1**. Prior to PSM, significant disparities existed among the control (n=1, 665), Kuntai (n=524), and GH (n=778) groups in several baseline parameters, including basal FSH, LH, AMH, AFC, as well as infertility type and causative factors (all p<0.05) (**Table 1**).

**Table 1:** Baseline characteristics of patients with PPOS ovulation induction before and after PSM.

	Before matching				After matching			
	Control group (n=1665)	Kuntai group (n=524)	GH group (n=778)	p	Control group (n=439)	Kuntai group (n=439)	GH group (n=439)	p
Age(years)	37.0 [32.0, 41.0]	36.0 [32.0, 40.0]	37.0 [33.0, 40.0]	0.151	36.0 [32.0, 40.0]	36.0 [32.0, 40.0]	36.0 [32.0, 39.0]	0.955
BMI (kg/m^2^)	22.0 [20.3, 24.2]	22.0 [20.3, 24.0]	22.0 [20.0, 23.8]	0.260	22.2 [20.4, 24.1]	22.2 [20.3, 24.0]	22.2 [20.1, 24.0]	0.950
Basic FSH (IU/L)	8.28 [6.43, 11.2]	9.02 [6.78, 12.3]	8.09 [6.36, 10.7]	<0.001	8.58 [6.62, 11.6]	8.64 [6.64, 11.7]	8.58 [6.86, 11.2]	0.963
Basic E_2_ (pg/ml)	34.0 [24.5, 49.0]	34.7 [24.3, 51.3]	37.0 [26.6, 52.2]	0.001	36.1 [27.0, 50.2]	35.2 [24.2, 51.5]	36.0 [26.4, 50.8]	0.544
Basic LH (IU/L)	3.70 [2.58, 5.29]	4.17 [2.97, 5.80]	3.82 [2.67, 5.26]	<0.001	3.94 [2.70, 5.42]	4.06 [2.89 5.57]	3.91 [2.80, 5.46]	0.643
AMH (ug/L)	0.60 [0.37, 0.87]	0.55 [0.30, 0.81]	0.68 [0.44, 0.97]	<0.001	0.56 [0.35, 0.78]	0.57 [0.31, 0.81]	0.57 [0.34, 0.85]	0.338
AFC	4.00 [3.00, 5.00]	4.00 [3.00, 5.25]	4.00 [3.00, 6.00]	0.001	4.00 [3.00, 5.00]	4.00 [3.00, 5.50]	4.00 [3.00, 6.00]	0.320
Infertility duration (years)	3.00 [2.00, 6.00]	3.00 [2.00, 6.00]	4.00 [2.00, 6.00]	0.037	4.00 [2.00, 6.00]	3.00 [2.00, 6.00]	4.00 [2.00, 6.00]	0.632
Infertility type, n (%)				0.021				0.936
Second	1131 (67.9%)	349 (66.6%)	567 (72.9%)		301 (68.6%)	299 (68.1%)	296(67.4%)	
Primary	534 (32.1%)	175 (33.4%)	211 (27.1%)		138 (31.4%)	140 (31.9%)	143 (32.6%)	
Infertility factors, n (%)				<0.001				0.770
Tubal factor	555 (33.3%)	161 (30.7%)	272 (35.0%)		144 (32.8%)	142 (32.3%)	136 (31.0%)	
Male factor	415 (24.9%)	124 (23.7%)	240 (30.8%)		107 (24.4%)	103 (23.5%)	96(21.9%)	
other	695 (41.7%)	239 (45.6%)	266 (34.2%)		188 (42.8%)	194 (44.2%)	207(47.2%)	

Continuous data are presented as median [IQR] and were compared using the Kruskal–Wallis test; categorical data are presented as percentages and were compared using the chi-square or Fisher’s exact test. PSM successfully balanced all baseline characteristics (*p* > 0.05 for all after matching). Other infertility factors include ovulation disorders, endometriosis, and uterine factors. Abbreviations: PSM, propensity score matching; BMI, body mass index; FSH, follicle-stimulating hormone; E_2_, estradiol; LH, luteinizing hormone; AMH, anti-Müllerian hormone; AFC, antral follicle count; GH, growth hormone.

After 1:1:1 propensity score matching, 439 patients were retained in each group, and all recorded baseline characteristics were well balanced, with no significant differences observed across the three groups for any variable (all *p* > 0.05, **Table 1**), confirming the effectiveness of the matching procedure in minimizing selection bias.

### Stimulation and embryological outcomes in the matched cohort

Analysis of the matched cohorts revealed distinct differences primarily attributable to GH pretreatment (**Table 2**). The GH group required a significantly higher total gonadotropin dose (median 1800 vs. 1500 IU in both other groups, adjusted p < 0.001) and exhibited a markedly higher serum E_2_ level on the trigger day (607 vs. 518 pg/mL in controls, adjusted p < 0.001), alongside lower LH and progesterone levels. This enhanced endocrine response translated into a significantly greater oocyte yield in the GH group (3.0 vs. 2.0 oocytes in controls, adjusted p < 0.001), as well as increased numbers of normally fertilized oocytes, cleaved embryos, and available embryos compared with both the control and Kuntai groups (all adjusted p < 0.05). However, the high-quality embryo rate on day 3 did not differ significantly across groups (approximately 34% in all groups, p = 0.948). The available embryo formation rate was higher in the GH group than in the Kuntai group (69.1% vs. 76.2%, adjusted p = 0.004).

**Table 2:** Ovulation induction outcomes and laboratory outcomes of DOR patients in each group after PSM.

	Control group (n=439)	Kuntai Group (n=439)	GH group (n=439)	*p*	Control VS kuntai		Control VS GH		Kuntai VS GH	
					*p* (unadj)	*p* (adj)	*p* (unadj)	*p* (adj)	*p* (unadj)	*p* (adj)
Total dose of Gn (U)	1500 [1200;1950]	1500 [1125;1950]	1800 [1350;2025]	<0.001	0.311	0.934	<0.001	<0.001	<0.001	<0.001
Dosing days of Gn (day)	8.00 [7.00;10.0]	9.00 [7.00;10.0]	8.00 [7.00;10.0]	0.330						
E_2_ on trigger day (pg/mL)	518 [355;776]	510 [334;728]	607 [388;956]	<0.001	0.119	0.354	<0.001	<0.001	<0.001	<0.001
LH on trigger day (IU/L)	3.00 [1.88;4.81]	3.25 [2.06;4.70]	2.91 [1.77;4.34]	0.029	0.090	0.271	0.092	0.276	0.004	0.011
P on trigger day (ng/mL)	0.20 [0.12;0.31]	0.19 [0.12;0.29]	0.19 [0.10;0.30]	0.034	0.050	0.149	0.005	0.015	0.178	0.533
Oocyte not obtained rate, n (%)	12 (2.73%)	22 (5.01%)	13 (2.96%)	0.134						
Number of oocytes retrieved	2.00 [1.00;4.00]	2.00 [1.00;3.00]	3.00 [2.00;4.00]	<0.001	0.020	0.061	<0.001	<0.001	<0.001	<0.001
Fertilization^*^, n (%)				0.162						
IVF	304 (72.0%)	278 (67.8%)	277 (66.1%)							
ICSI or rescue icsi	118 (28.0%)	132 (32.2%)	142 (33.9%)							
Number of normally fertilized embryos^*^	2.00 [1.00;3.00]	2.00 [1.00;2.00]	2.00 [1.00;3.00]	<0.001	0.392	1.000	<0.001	<0.001	<0.001	<0.001
Number of cleavages in normally fertilized embryos^*^	2.00 [1.00;2.00]	2.00 [1.00;2.00]	2.00 [1.00;3.00]	<0.001	0.493	1.000	<0.001	<0.001	<0.001	<0.001
Number of high-quality embryos on day 3^*^	0.00 [0.00;1.00]	0.00 [0.00;1.00]	1.00 [0.00;1.00]	0.021	0.449	1.000	0.007	0.020	0.010	0.029
number of available embryos^*^	1.00 [1.00;2.00]	1.00 [1.00;2.00]	2.00 [1.00;2.00]	0.004	0.239	0.717	<0.001	0.002	0.007	0.020
Normal fertilization rate^*^, n (%)	813 (68.4%)	754 (70.3%)	960 (67.2%)	0.267						
Normal cleavage rate^*^, n (%)	790 (97.2%)	740 (98.1%)	944(98.3%)	0.202						
high-quality embryos on day 3 rate^*^, n (%)	266 (33.7%)	255 (34.5%)	321(34.0%)	0.948						
Available embryos formation rate^*^, n (%)	564 (71.4%)	564 (76.2%)	652 (69.1%)	0.005	0.037	0.111	0.317	0.951	0.001	0.004
unusable embryos rate^*^, n (%)	103 (24.4%)	79 (19.3%)	83 (19.8%)	0.135						

Data presentation and statistical comparisons followed the methods described in **Table 1**. *Post-hoc* pairwise comparisons were performed using Dunn’s test with Bonferroni correction. Embryological variables marked with * were calculated from 422, 410, and 419 cycles in the control, Kuntai, and GH groups, respectively, due to exclusion of cycles with no retrieved oocytes or failed fertilization. Additional abbreviations: Gn, gonadotropins; P, progesterone; IVF, *in vitro* fertilization; ICSI, intracytoplasmic sperm injection.

In contrast, the Kuntai group showed no statistically significant improvement over the control group in any ovarian response or embryological parameter after Bonferroni correction, despite a trend toward a lower unusable embryo rate that did not reach statistical significance (adjusted p= 0.111).

### Pregnancy and live birth outcomes following FET

Endometrial thickness and embryo developmental stage at transfer were similar across three groups (p > 0.05). However, the distribution of endometrial preparation protocols differed significantly (p=0.002), with the Kuntai group employing artificial cycles more frequently (33.3%) than the control group (22.1%, adjusted p=0.046, **Table 3**).

**Table 3:** Frozen-thawed embryo transfer clinical outcomes of DOR patients in each group after PSM.

	Control group (n=249)*	Kuntai group (n=240) *	GH group (n=238)*	*p*	Control VS kuntai		Control VS GH		Kuntai VS GH	
					*p* (unadj)	*p* (adj)	*p* (unadj)	*p* (adj)	*p* (unadj)	*p* (adj)
Endometrial preparation protocol, n (%)				0.002	0.015	0.046	0.108	0.325	0.004	0.012
Artificial cycle	55 (22.1%)	80 (33.3%)	58 (24.4%)							
Downregulated artificial cycle	159 (63.9%)	124 (51.7%)	161 (67.6%)							
Other **	35 (14.1%)	36 (15.0%)	19 (7.98%)							
Endometrial thickness (mm)	9.20 [8.30, 10.7]	9.20 [8.17, 10.4]	9.20 [8.40, 10.3]	0.865						
No. of embryos transferred	2.00 [2.00;2.00]	2.00 [1.00;2.00]	2.00 [2.00;2.00]	0.004	0.001	0.003	0.370	1.000	0.003	0.009
Stage of embryos transferred, n (%)				0.226						
Cleavage	215 (86.3%)	194 (80.8%)	195 (81.9%)							
Other ***	34 (13.7%)	46 (19.2%)	43 (18.1%)							
Biochemical pregnancy rate, n (%)	142 (57.3%)	128 (53.3%)	159 (67.4%)	0.006	0.435	1.000	0.028	0.083	0.002	0.007
Implantation rate, n (%)	132 (29.0%)	128 (31.4%)	127 (29.7%)	0.744						
Clinical pregnancy rate, n (%)	110 (44.5%)	110 (45.8%)	107 (45.7%)	0.950						
Pregnancy loss rate, n (%)	26 (28.9%)	27 (31.8%)	21 (26.9%)	0.791						
Live birth rate, n (%)	64 (28.2%)	58 (27.0%)	57 (27.8%)	0.959						

Data presentation, statistical methods and *post-hoc* pairwise comparisons are as described in **Tables 1**, **2**. *Thawing cycles (some patients had >1 cycle). **Natural and ovulation induction cycles combined as “Other”. ***D5/D6 blastocysts and sequential transfers combined as “Other”.

A significant intergroup difference was observed in the biochemical pregnancy rate (p=0.006), with the GH group achieved a higher rate (67.4%) than both the control (57.3%, unadjusted p=0.028) and Kuntai groups (53.3%, adjusted p=0.007). Nevertheless, this early advantage did not persist. Clinical pregnancy rates (44.5%- 45.8%) and live birth rates (27.0%- 28.2%) were statistically indistinguishable. Pregnancy loss rates were also comparable (27%- 32%, p=0.791).

Although the Kuntai group received slightly fewer embryos per transfer (median 2.00 vs. 2.00, adjusted p= 0.003), implantation rates remained consistent across groups (29.0%–31.4%, p= 0.744).

### Multivariate analysis using GEE models

GEE models, adjusting for confounders, confirmed the null effect of the adjuvants on key outcomes (**Table 4**). Compared with the control group, neither Kuntai capsule nor GH pretreatment was significantly associated with biochemical pregnancy (adjusted OR 0.77, 95% CI 0.53–1.12, p = 0.176; and adjusted OR 1.40, 95% CI 0.95–2.06, p = 0.090, respectively), clinical pregnancy (adjusted OR 0.99, 95% CI 0.68–1.44, p = 0.942; and adjusted OR 0.93, 95% CI 0.63–1.36, p = 0.691), or live birth (adjusted OR 0.90, 95% CI 0.58–1.39, p = 0.636; and adjusted OR 0.85, 95% CI 0.54–1.33, p= 0.480).

**Table 4:** Multivariate logistic regression GEE model with odds ratios for frozen-thawed embryo transfer biochemical pregnancy, clinical pregnancy and live birth in three groups.

	Biochemical pregnancy		Clinical pregnancy		Live birth	
	aOR (95% CI)	*p*	aOR (95% CI)	*p*	aOR (95% CI)	*p*
Treatment						
Control (reference)						
kuntai	0.77 (0.53, 1.12)	0.176	0.99 (0.68, 1.44)	0.942	0.90 (0.58, 1.39)	0.636
GH	1.40 (0.95, 2.06)	0.09	0.93 (0.63, 1.36)	0.691	0.85 (0.54, 1.33)	0.480
Age (year)						
<35 (reference)						
35-37	1.25 (0.79, 1.98)	0.333	0.89 (0.58, 1.36)	0.584	0.91 (0.57, 1.47)	0.70
>37	0.54 (0.38, 0.76)	0.000	0.42 (0.30, 0.60)	0.000	0.36 (0.23 0.55)	0.000
BMI (kg/m^2^)						
<18.5 (reference)						
18.5-24	1.59 (0.88, 2.85)	0.124	1.80 (1.00, 3.25)	0.052	2.28 (1.03, 5.04)	0.043
>24	1.71 (0.89, 3.27)	0.104	2.11 (1.11, 4.04)	0.024	2.56 (1.09, 6.01)	0.032
Endometrial preparation protocol						
Artificial cycle (reference)						
Downregulated artificial cycle	1.35 (0.93, 1.95)	0.111	1.45 (1.00, 2.08)	0.047	1.15 (0.76, 1.74)	0.505
Other *	0.90 (0.53, 1.53)	0.695	0.75 (0.43, 1.32)	0.322	0.80 (0.41, 1.57)	0.520
Reason						
Tubal factor (reference)						
Male factor	0.69 (0.46, 1.06)	0.088	0.62 (0.40 0.94)	0.023	0.73 (0.44, 1.20)	0.213
Other factor **	0.88 (0.62, 1.26)	0.483	0.75 (0.52, 1.06)	0.099	0.87 (0.58 1.29)	0.480
No. of embryos transferred	1.07 (0.70, 1.64)	0.75	1.08 (0.73, 1.61)	0.688	1.49 (0.99, 2.23)	0.005
Phase of embryo transferred (D3 vs. Other) ***	3.53 (1.91, 6.50)	0.000	2.34 (1.37, 4.00)	0.002	1.75 (0.96, 3.16)	0.066

* Other endometrial preparation: natural and ovulation induction cycles. ** Other infertility factors: ovulation disorders, endometriosis, uterine factors, etc. *** Other embryo stage: D5/D6 blastocysts and sequential transfers. Additional abbreviations: aOR, Adjusted odds ratios.

Patient age emerged as a powerful, independent predictor of success. Women over 37 years of age had substantially reduced odds of achieving a biochemical pregnancy (adjusted OR 0.54, 95% CI 0.38-0.76; p<0.001), clinical pregnancy (adjusted OR 0.42, 95% CI 0.30-0.60; p<0.001), and live birth (adjusted OR 0.36, 95% CI 0.23-0.55; p<0.001) compared to their counterparts under 35 years. Body mass index (BMI) also showed a significant association. Patients with a BMI >24 kg/m^2^ had higher odds of clinical pregnancy (adjusted OR 2.11, 95% CI 1.11-4.04; p=0.024) and live birth (adjusted OR 2.56, 95% CI 1.09-6.01; p=0.032) compared to underweight women (BMI <18.5 kg/m^2^). Furthermore, the use of a down-regulated artificial cycle protocol was associated with improved clinical pregnancy rates relative to the standard artificial cycle (adjusted OR 1.45, 95% CI 1.00-2.08; p=0.047).

### Sensitivity analysis

For the primary outcome (oocyte yield), the observed effect size (Cohen’s f = 0.222) with the current sample size (n = 439 per group) provided 100% power, and only 66 patients per group would be required to achieve 80% power. This confirms that our sample was more than sufficient to reliably detect the superiority of GH in increasing oocyte yield, supporting our confidence that GH genuinely improves oocyte retrieval in DOR patients.

In contrast, for secondary clinical outcomes including clinical pregnancy, pregnancy loss, and live birth, the absolute differences among the three groups were minimal (clinical pregnancy: 44.5% vs. 45.8% vs. 45.7%; live birth: 28.2% vs. 27.0% vs. 27.8%), with correspondingly small effect sizes (w = 0.012–0.043). The current sample provided only 5.6–8.7% power to detect such minimal differences; achieving 80% power would require approximately 22, 000–25, 000 patients per group—clearly beyond the scope of a single-center study. Therefore, while our data do not support a significant benefit of GH or Kuntai capsule in improving live birth rates, these null findings should be interpreted in the context of the extremely small effect sizes observed (**Supplementary Table 1**).

## Discussion

This large, matched-cohort study yielded two key findings regarding the use of GH and Kuntai capsule as adjuvants in DOR patients undergoing PPOS with a freeze-all strategy. First, GH supplementation significantly improved ovarian response and oocyte yield, but this benefit did not translate into superior clinical outcomes. Second, Kuntai capsule pretreatment showed no measurable improvement in any ovarian, embryological, or clinical outcomes.

The most salient finding was the clear dissociation between GH’s effects on the stimulation phase and the final treatment outcome. GH significantly augmented ovarian responsiveness, evidenced by higher E_2_ levels(607 vs 518 pg/mL, p<0.001), oocyte yield(3 vs 2, p<0.001), and embryo numbers—an endocrine and laboratory profile suggesting improved follicular recruitment and function. This endocrine profile - characterized by elevated E_2_ with appropriately suppressed LH and progesterone levels - suggests GH may optimize follicular microenvironment by enhancing granulosa cell function while preventing premature luteinization. These findings are consistent with the existing literature suggesting that growth hormone may enhance granulosa cell sensitivity and responsiveness to gonadotropin or growth hormone stimulation, thereby modulating sex steroid synthesis and follicular development, and improve oocyte quality, embryo competence (25, 26). Although the GH group received a higher total Gn dose, this likely reflects the individualized dosing strategy in response to slower follicular development, rather than a protocol-driven difference, and the multivariate GEE models were adjusted for key confounders including age and baseline ovarian reserve parameters. However, the critical observation is that this early advantage, including a higher biochemical pregnancy rate, did not propagate into improved clinical pregnancy or live birth. Our multivariate GEE model solidly confirmed that GH was not an independent predictor of these ultimate success measures (live birth adjusted OR 0.85, p=0.48).

Several factors contribute to the ongoing controversy regarding GH’s efficacy on clinical pregnancy and live birth outcomes (27–30). First, substantial variations exist in GH administration protocols, including differences in treatment initiation (ranging from during ovarian stimulation to 2–6 weeks pretreatment) and dosage (2-7.5 IU vs. 12–24 IU) (31). Second, the use of different COS protocols (GnRH antagonist vs. agonist regimens) may lead to inconsistent responses (20). A compelling hypothesis, particularly relevant to our study design, is that GH’s primary mechanism may be endometrial rather than follicular (32). A freeze-all strategy, by physically separating the stimulated cycle from the embryo transfer, effectively negates any potential endometrial benefit of GH, leaving only its modest ovarian effects which appear insufficient to influence the ultimate outcome of live birth. Additionally, GH’s efficacy is age-dependent, yielding significant improvements in embryo quality and live birth rates exclusively in DOR patients aged 35–40 years, with no comparable effects observed in younger (<35) or older (>40) patients (21). Thus, within a freeze-all paradigm, the modest ovarian effects of GH appear insufficient to overcome the primary barrier of oocyte quality in DOR, which is predominantly age-driven.

Contrary to our initial hypothesis and some previous reports, Kuntai capsule pretreatment demonstrated a notable absence of reproductive benefits in our cohort. We observed no significant enhancement in ovarian responsiveness (trigger-day E2: 510 [334;728] vs control 518 [355;776] IU/L, adjusted p=0.354), oocyte yield (2.00 [1.00-3.00] vs 2.00 [1.00-4.00], adjusted p=0.061), or embryo quality (high-quality embryo rate: 34.5% vs 33.7%, p=0.948). Also, we detected no improvement in the ultimate endpoints of clinical pregnancy (45.8% vs 44.5%, p=0.950) or live birth (27.0% vs 28.2%, p=0.959). The GEE model corroborated these findings, indicating that Kuntai was not an independent factor influencing clinical pregnancy (adjusted OR 0.99, 95% CI 0.68-1.44, p=0.942) or live birth (adjusted OR 0.90, 95% CI 0.58-1.39, p=0.636). The existing evidence base for Kuntai in improving IVF/ICSI outcomes for DOR is limited and appears context-dependent. Previous reports (12, 15, 16), including two randomized controlled trials (RCT) (16, 17), have suggested that Kuntai capsule may improve ovarian response and clinical outcomes in DOR patients. However, these studies differed fundamentally from our design: they enrolled smaller cohorts (n=108 and n=70), administered pretreatment over three menstrual cycles, and exclusively employed fresh embryo transfer protocols. The disparity in outcomes underscores that any potential benefits of Kuntai may be negated within the specific framework of a PPOS-freeze-all strategy.

Beyond the interventions, our regression analysis underscores the dominant role of patient-specific factors. The profound negative impact of advanced maternal age (>37 years) on live birth (adjusted OR 0.36, p<0.001) reiterates the irreversible influence of chronological age on oocyte competence in DOR patients (33–35). While previous studies have predominantly emphasized the reproductive risks associated with overweight BMI (36, 37), our analysis revealed a more nuanced relationship. Surprisingly, patients with ultra-low BMI (<18.5 kg/m^2^) demonstrated the poorest outcomes, showing significantly lower live birth rates compared to both normal-weight (adjust OR 2.28, 95% CI 1.03-5.04, p=0.043) and overweight groups (adjust OR 2.56, 95% CI 1.09-6.01, p=0.032). This pattern held consistently across clinical pregnancy rates (BMI 18.5-24: adjust OR 1.80, p=0.052; BMI >24: adjust OR 2.11, p=0.024) and biochemical pregnancy outcomes. Notably, the detrimental effects of low BMI exceeded those of overweight status across all reproductive endpoints.

*Post-hoc* power analysis demonstrated 100% power for detecting the observed difference in oocyte yield, further supporting that GH genuinely improves oocyte retrieval, while Kuntai capsule does not. For clinical pregnancy, pregnancy loss, and live birth, the observed differences were minimal, with correspondingly low power (5.6–5.8%), suggesting that any potential benefit of these adjuvants on clinical outcomes is likely limited.

The limitations of our study, including its retrospective design and single-center nature, necessitate validation through prospective, multicenter trials. Furthermore, the heterogeneity in adjuvant administration protocols—specifically, the shorter treatment duration in our center compared with longer pretreatment regimens reported in some previous studies—should be considered when interpreting and generalizing our findings. In addition, the sample size, while large overall, was insufficient for robust subgroup analyses stratified by age, endometrial preparation protocols, or embryo stage at transfer. Additionally, the retrospective nature and individualized Gn dosing may introduce residual confounding, as the GH group received higher total gonadotropin doses, though this likely reflects slower follicular response rather than a systematic bias. Future research should prioritize prospective designs that can further elucidate the role of these and other adjuvants within specific DOR subpopulations and treatment protocols.

## Data Availability

The data analyzed in this study is subject to the following licenses/restrictions: Data cannot be publicly shared due to patient privacy protection and ethical restrictions imposed by the ethics committee (Approval No. SZYY-202504). Reasonable requests for de-identified data will be considered by the corresponding author, pending ethics committee approval. Requests to access these datasets should be directed to JT tanjun561127@163.com.
