# Supplementary material for: Growth hormone enhances ovarian response but not live births in DOR patients undergoing PPOS with freeze-all strategy: a retrospective cohort study contrasted with Kuntai capsule
Source: Front Endocrinol (Lausanne). 2026 Aug 3;17:1897970. doi: 10.3389/fendo.2026.1897970 (PMC13477937; doi:10.3389/fendo.2026.1897970)
Supplement: Supplementary file 1 [file Table1.docx]

Table S1. Post-hoc power analysis for key endpoints

| Endpoint | Group rates / values | | | Effect size | Power (%) | N needed per group (80% power) |
| --- | --- | --- | --- | --- | --- | --- |
|  | Control | Kuntai | GH |  |  |  |
| Primary outcome | | | | | | |
| Oocytes retrieved, median [IQR] | 2.0 [1.0–4.0] | 2.0 [1.0–3.0] | 3.0 [2.0–4.0] | f = 0.222 | 100.0 | 66 |
| Secondary clinical outcomes | | | | | | |
| Clinical pregnancy, % (n) | 44.5 (247) | 45.8 (240) | 45.7 (234) | w = 0.012 | 5.8 | 22,636 |
| Pregnancy loss, % (n) | 28.9 (90) | 31.8 (85) | 26.9 (78) | w = 0.043 | 8.7 | 1,731 |
| Live birth, % (n) | 28.2 (227) | 27.0 (215) | 27.8 (205) | w = 0.011 | 5.6 | 24,556 |

Notes: Effect sizes: Cohen's f for ANOVA (oocytes retrieved); Cohen's w for chi-square tests (clinical outcomes). Power was calculated with α = 0.05 (two-sided). N needed per group indicates the sample size required per group to achieve 80% power to detect the observed effect size.
